# *Pdcd1^−/−^* Mice Exhibit High Susceptibility to Respiratory Syncytial Virus Infection

**DOI:** 10.1155/jimr/1223280

**Published:** 2026-10-22

**Authors:** Chenyu Zhang, Weina Guan, Yi Zhang, Yao Zhang, Qiuhong Li, Xin Sun, Ruixue Ma

**Affiliations:** ^1^ Department of Pediatrics Xijing Hospital The Fourth Military Medical University Xi’an China fmmu.edu.cn

**Keywords:** animal model, neutrophil, *Pdcd1*, RSV, Th2 response

## Abstract

**Background:**

Respiratory syncytial virus (RSV) is a major cause of severe lower respiratory tract illness in infants, yet no highly effective treatment exists. The widely used BALB/c mouse model is only semipermissive to RSV and does not fully recapitulate the severe disease seen in humans, highlighting the need for improved preclinical models.

**Methods:**

WT and *Pdcd1* ^*−/−*^ mice were challenged intranasally (i.n.) with a low dose of RSV (10^6^ PFU). Disease severity was assessed by weight loss, lung function, viral load, and histopathology. Immune responses were characterized via flow cytometry, immunohistochemistry, and cytokine/chemokine measurement.

**Results:**

Compared to WT mice, *Pdcd1* ^*−/−*^ mice developed significantly more severe illness, evidenced by exacerbated weight loss, lung dysfunction, histopathology, and elevated viral replication. The acute phase was marked by robust neutrophil lung infiltration, elevated chemokines (CXCL1 and CXCL2), and heightened myeloperoxidase (Mpo) activity. *Pdcd1* knockout skewed the adaptive immune response, promoting a Th2‐biased CD4^+^ T cell infiltration while impairing CD8^+^ T cell cytotoxicity, as indicated by reduced perforin and granzyme B expression. Critically, *Pdcd1* ^*−/−*^ mice exhibited a profound defect in antiviral cytokine production, with significantly lower levels of both IFN‐γ and IFN‐α in lungs and serum postinfection.

**Conclusions:**

*Pdcd1* knockout renders mice highly susceptible to RSV, manifesting as enhanced viral replication, severe immunopathology featuring Th2 bias and neutrophil‐driven inflammation, and a blunted antiviral interferon response. This phenotype more closely mirrors key aspects of severe human RSV infection, establishing the *Pdcd1* ^*−/−*^ mouse as a sensitive animal model for evaluating novel therapeutics.

## 1. Introduction

Respiratory syncytial virus (RSV) remains a leading global cause of acute lower respiratory tract infections, posing a substantial burden on infants, young children, and immunocompromised individuals [1]. Each year, ~33 million cases of lower respiratory tract infections caused by RSV are recorded among children younger than 5 years. This leads to an estimated 3.6 million hospital admissions and about 118,200 fatalities globally [2]. Despite decades of research, effective therapeutic interventions remain limited [3], and vaccine development has faced considerable challenges. This underscores the critical need for a deeper understanding of RSV immunopathogenesis and the development of reliable preclinical models that faithfully recapitulate the human disease spectrum.

A major limitation in RSV research is the lack of a fully suitable animal model [4]. While inbred mice, particularly the BALB/c strain, are widely used, they are only semipermissible to RSV [5]. To elicit airway hyperresponsiveness, these models require an intranasally (i.n.) inoculated dose as high as 10^7^ PFU [6], far exceeding the 1000 PFU sufficient to cause symptomatic infection in humans [7]. Mouse models often fail to replicate the severe lower respiratory tract pathology seen in high‐risk populations like infants. This disparity in disease severity hinders the translational evaluation of novel therapeutics and vaccines, underscoring the urgent need to develop more susceptible and immunologically relevant animal models.

Programmed cell death 1 (PD‐1), a key immune checkpoint receptor, plays a pivotal role in modulating T‐cell activation, tolerance, and exhaustion to maintain immune homeostasis [8]. Its function has been extensively studied in the contexts of chronic viral infections, cancer, and autoimmunity [9]. In respiratory viral infections, PD‐1 signaling is thought to fine‐tune the immune response, preventing excessive immunopathology [10]. However, its specific role in acute RSV infection, particularly in shaping the early innate and adaptive immune landscape, remains poorly defined. Intriguingly, clinical observations suggest that dysregulated or insufficient immune responses may drive severe RSV disease in young children [11], hinting at a potential role for immune regulatory pathways like PD‐1.

We hypothesized that *Pdcd1* knockout would disrupt the delicate balance of antiviral immunity during RSV infection, leading to enhanced disease severity. To test this, we employed a *Pdcd1* ^*−/−*^ mouse model challenged with a low‐dose RSV inoculum (10^6^ PFU per mouse). We found that *Pdcd1* ^*−/−*^ mice exhibit markedly heightened susceptibility to RSV, manifesting as exacerbated disease and pathological outcomes. Mechanistically, the severe phenotype was driven by a dysregulated immune response featuring robust neutrophilic inflammation, a Th2‐skewed T‐cell profile, impaired cytotoxicity, and blunted interferon production, which mirrors several key aspects of severe RSV infection in human infants. Thus, our work establishes *Pdcd1* ^*−/−*^ mice as a novel and sensitive preclinical model for evaluating future RSV countermeasures.

## 2. Materials and Methods

### 2.1. Virus Stocks

RSV strains long (ATCC VR‐26) and A2 (ATCC VR‐1540) were used for mouse challenge experiments. HeLa cells served as RSV‐sensitive cell lines and were used for virus propagation [12]. The determination of viral infection titers was conducted via plaque assays.

### 2.2. Generation of *Pdcd1* Knockout Mouse Model

For obtaining a *Pdcd1* knockout (*Pdcd1* ^*−/−*^) mouse, Cas9 mRNA and gRNA were prepared by in vitro transcription. Two pairs of gRNA were targeted to delete exons 2–4 of the *Pdcd1* gene (Figure S1A). Zygotes of BALB/c mice were received, injected with in vitro‐transcribed Cas9 mRNA and sgRNAs, and transferred to pseudopregnant recipients. The genotype of the obtained F0 mice was identified and confirmed by PCR. The primer sequences used in the genotyping are listed in Table S1. By crossing with BALB/c mice, positive F0 mice were chosen for obtaining F1 heterozygous *Pdcd1* ^*−/−*^ mice. For obtaining the homozygous *Pdcd1* ^*−/−*^ mice, female and male F1 heterozygous mice were intercrossed randomly. Mice aged 6–8 weeks were used for all studies.

### 2.3. Animal Infection

Mice were i.n. inoculated with 10^6^ PFU of RSV in 60 μL of PBS (Plant Chem Medicine, PC‐00003, China). Control mice were given an equivalent volume of sterile PBS, UV‐inactivated RSV, and HeLa cell lysate. Body weight and survival were monitored daily.

### 2.4. Assessment of Pulmonary Function

The pulmonary function of mice was evaluated using a whole‐body plethysmography (WBP) system (TOW‐INT TECH, China), following the manufacturer’s instructions. Briefly, unrestricted, unanesthetized mice were housed in Plexiglas chambers for 60 min per day over 3 days to acclimate to the environment. During formal testing, the chambers were connected to a sensor probe. Fluctuations of the mouse chest caused changes in the chamber’s volume. Respiratory parameters were recorded for 30 min to establish a baseline, and only data collected while the animals were quietly resting awake were included in the analysis. Enhanced pause (Penh) was calculated and averaged over a 10‐min period.

### 2.5. Plaque Assay

Whole lungs were harvested from mice, weighed, mechanically homogenized, and supernatants were separated by centrifugation at 10,000 × *g* for 10 min. HeLa cells were infected with 100 μL of serially diluted RSV‐infected lung samples (10‐fold dilutions) for 2 h at 37°C. Wells were overlaid with 1 × DMEM/1% methylcellulose. After incubating at 37°C and 5% CO_2_ for 5 days, the supernatants were carefully removed. The cells were fixed and subsequently stained with 1% crystal violet in methanol. Plates were washed with water and allowed to dry overnight, and plaques were counted the next morning.

### 2.6. ELISA Assay

The concentrations of IFN‐γ and IFN‐α in lung tissues from mice were measured using commercial ELISA kits (NEOBIOSCIENCE, China) according to the manufacturer’s instructions. Briefly, 100 μL aliquots of lung homogenates were added to the precoated 96‐well plates. The absorbance was read at 450 nm using a BioTek Synergy HTX Multi‐Mode Microplate Reader (Agilent, USA). Protein concentrations were interpolated from the standard curve of each kit, and all samples were assayed in triplicate.

### 2.7. Quantitative Real‐Time PCR and Absolute Quantification of Viral Load

Whole lungs were harvested, weighed, and mechanically homogenized by an automated homogenizer‐TissueLyser II (QIAGEN, Germany). For total RNA extraction, 200 μL of tissue homogenates was transferred to 500 μL of TRIzol reagent, followed by chloroform extraction and isopropanol precipitation. Finally, RNA was redissolved in RNase‐free water. cDNA was synthesized by using the Evo M‐MLV RT Premix for quantitative PCR (qPCR) (Accurate Biotechnology, AG11706, China). qPCR was performed using the SYBR Green Premix Pro Taq HS qPCR Kit (Accurate Biotechnology, AG11701, China) on a LightCycler 96 (Roche, Switzerland). The primer sequences used in this study are listed in Table S2.

For absolute quantification of viral load, the full‐length RSV N gene was cloned into the pCAGGS vector to generate a standard. Tenfold serial dilutions of the standard, ranging from 10^3^ to 10^10^ copies per reaction, were used to construct a standard curve, which yielded a slope of − 3.5942, a *Y*‐intercept of 40.385 cycles, and an *R* ^2^ value of 0.9994. Viral copy numbers in each sample were determined by interpolating the *C*_*q*_ values onto this standard curve.

### 2.8. Histology Analysis

Lung tissues were fixed in 4% paraformaldehyde and sectioned at 5 μm. For IHC staining, sections were incubated with primary antibodies against Ly6g (GB11229−100, Servicebio, China) and CD3 (GB13014−50, Servicebio, China), followed by HRP‐conjugated secondary antibody (GB23303, Servicebio, China), and visualized with DAB. Images were captured using a 3DHISTECH scanner and analyzed with CaseViewer 2.4. Six randomly selected nonoverlapping fields per section were quantified using Image J, and the results are expressed as the percentage of positive cells per field. All analyses were performed in a blinded manner. H&E staining was performed following standard protocols [13]. Histological images were acquired using the same system. The degree of lesions was visually scored using a pre‐established 4‐grade system: 0 for normal, 1 for minimal, 2 for mild, 3 for moderate, and 4 for severe [14].

### 2.9. Isolation of Cells From Lung and Bronchoalveolar Lavage (BAL)

Mice were euthanized, and cells were harvested from the airways. BAL was performed by instilling and withdrawing 1 mL of PBS three times. The collected BAL fluid was centrifuged at 1500 × rpm for 10 min to pellet the cellular fraction.

Lung tissues were carefully dissected from the mice, minced into small fragments, and collected in tubes containing a digestion solution prepared with PBS supplemented with 10% fetal bovine serum (FBS, PCM, PC‐00001, China). This digestion solution consisted of 1 mg/mL Collagenase I (BioFroxx, 1904MG100, China) and 0.25 mg/mL DNase I (Biosharp, BS137–10 mg, China). The samples were then incubated for 30 min at 37°C. Following digestion, the tissue suspension was filtered through a 70 μm nylon mesh. The filtrate was collected to obtain a single‐cell suspension of lung tissue. To remove red blood cells (RBC), the cell suspension was treated with a commercial RBC lysis buffer (Biosharp, BL503B, China). Finally, the cells were pelleted by centrifugation, resuspended in flow cytometry staining buffer (PBS containing 2% FBS), and counted.

### 2.10. Flow Cytometry

Isolated lung and BALF cells were adjusted to 10^6^ cells/mL and stimulated with cell activation cocktail (with brefeldin A) (BioLegend, 423303, USA) for 4 h at 37°C. After stimulation, cells were harvested, filtered through a 70‐μm strainer, and resuspended at 10^6^ cells per tube for flow cytometric staining. Cells were first blocked with the anti‐CD16/CD32 antibody (BioLegend, 156604, USA) on ice for 10 min. For surface staining, single‐cell suspensions were incubated with the following fluorochrome‐conjugated antibodies (Biolegend, USA) for 30 min at 4°C: Alexa Fluor 700 anti‐mouse CD45, BV605 anti‐mouse CD45, FITC anti‐mouse CD11b, BV785 anti‐mouse Ly6g, FITC anti‐mouse CD3, PE anti‐mouse CD4, PerCP/Cy5.5 anti‐mouse CD4, and APC/Cy7 anti‐mouse CD8a. For intracellular cytokine staining, cells were fixed and permeabilized with BD Cytofix/Cytoperm solution (250 μL/tube) for 20 min at 4°C. After two washes with 1 × perm/wash buffer (BD, 554714, USA), cells were stained with antibodies against BV421 anti‐mouse IFN‐γ, APC anti‐mouse granzyme B, PE anti‐mouse perforin, and APC anti‐mouse IL‐4 (all from Biolegend, USA). Stained cells were resuspended in 400 μL PBS and acquired on a BD FACSCelesta flow cytometer (USA). Data analysis was performed using FlowJo software (Version 10.8.1). The gating strategies are shown in Figures S2–S4.

### 2.11. Animal Subject Studies

All animal experiments were approved by the Medical Ethics Committee of the First Affiliated Hospital of the Air Force Medical University (Number 20250076).

### 2.12. Statistics

All data were presented as the mean ± the standard deviation (SD) based on a minimum of three independent experiments. Statistical methods are specified in the figure legends, and all analyses were carried out with GraphPad Prism 9 software. Significant values were indicated as  ^∗^ *p* < 0.05,  ^∗∗^ *p* < 0.01,  ^∗∗∗^ *p* < 0.001 or  ^∗∗∗∗^ *p* < 0.0001 in the figures.

## 3. Results

### 3.1. *Pdcd1* ^*−/−*^ Mice Exhibit Increased Susceptibility to Intranasal RSV Long Strain Infection

To elucidate the role of PD‐1 in RSV pathogenesis, we generated *Pdcd1* ^*−/−*^ mice (Figure S1) and subjected both WT and *Pdcd1* ^*−/−*^ mice to an intranasal challenge with the RSV Long strain. Two additional control groups were included: UV‐inactivated RSV and the HeLa cell lysate. Body weight was monitored daily throughout the course of the infection. In WT mice, no significant differences in body weight changes were observed among the RSV, UV‐inactivated RSV, and HeLa cell lysate groups. In contrast, *Pdcd1* ^*−/−*^ mice infected with active RSV exhibited a marked and progressive weight loss compared with that of the two control groups. Notably, at 5 dpi, *Pdcd1* ^*−/−*^ mice had lost ~10.7% of their baseline body weight, whereas WT mice showed no significant change. Overall, the difference in weight changes between the two genotypes was statistically significant (*p* < 0.01; Figure 1A). Airway function was assessed by measuring Penh, which reflects changes in the breathing pattern. After the RSV challenge, pulmonary dysfunction was significantly more severe in *Pdcd1* ^*−/−*^ mice compared to WT controls (*p* < 0.05; Figure 1B). Histopathological examination of lung tissues at 4 and 7 dpi revealed more extensive perivascular and alveolar wall infiltration of inflammatory cells in infected *Pdcd1* ^*−/−*^ mice (Figure 1C,D). We next evaluated the RSV replication kinetics in the lungs. Viral RNA was detectable from 2 to 8 dpi. Notably, the viral load in *Pdcd1* ^*−/−*^ mice was significantly higher than in WT mice at 2, 4, and 6 dpi (*p* < 0.0001, Figure 1E). Consistent with this, plaque‐forming assays demonstrated that lung viral titers were significantly elevated in *Pdcd1* ^*−/−*^ mice at both 4 dpi (*p* < 0.05) and 6 dpi (*p* < 0.05) (Figure 1F). Collectively, these findings indicated that *Pdcd1* knockout enhances susceptibility to the RSV infection.

**Figure 1:**
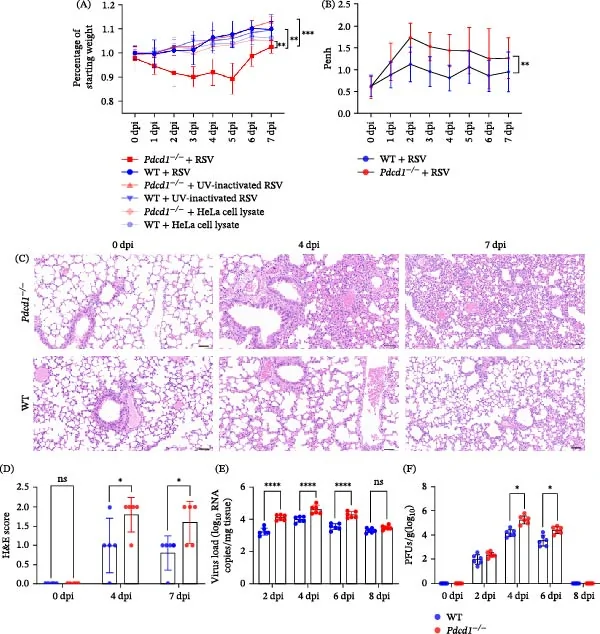
*Pdcd1* ^*−/−*^ mice are susceptible to intranasal RSV infection. Mice were inoculated intranasally with 10^6^ PFU of purified RSV long strain. Two control groups were included: UV‐inactivated RSV and HeLa cell lysate. (A) Body weight was monitored daily for 7 days postinfection (dpi). At the time of inoculation (0 dpi), the body weights of mice were set as the baseline, and subsequent weight changes relative to the baseline are shown (*n* = 3–5 mice per group). (B) The enhanced pause (Penh) was measured daily up to 7 dpi for *Pdcd1* ^*−/−*^ and WT mice using whole‐body plethysmography (*n* = 8 mice per group). (C) Lungs were collected on 4 and 7 dpi and subjected to H&E staining. Scale bars, 50 μm. (D) Statistical analysis based on histological scores for perivascular and alveolar wall inflammatory cell infiltration. *n* = 5 mice per group. (E) Viral loads in the lungs of RSV‐infected mice were measured at the indicated time points. *n* = 6 mice per group. (F) Virus titers in the lung tissue of RSV‐infected mice were measured by plaque assay and expressed as plaque forming units per gram (PFUs/g, *n* = 6 mice per group). The data presented represent a representative experiment. All data are presented as mean ± SD. Statistical significance was determined by two‐way ANOVA with Tukey’s post hoc test.  ^∗^ *p* < 0.05,  ^∗∗^ *p* < 0.01,  ^∗∗∗∗^ *p* < 0.0001 versus WT mice; ns, not significant.  ^∗∗∗^ indicate statistical significance (*p* < 0.001).

### 3.2. *Pdcd1* ^*−/−*^ Mice Exhibited Increased Neutrophil Infiltration in the Lungs During Acute Virus Infection

Neutrophil recruitment is an early and prominent feature of human RSV infection and is positively correlated with disease severity [15]. To investigate whether the exacerbated disease phenotype in *Pdcd1* ^*−/−*^ mice is linked to neutrophil responses, we quantified pulmonary neutrophils at various time points postinfection. Flow cytometric analysis of lung cell suspensions at 0, 2, 4, and 7 dpi revealed that neutrophil counts were significantly elevated in *Pdcd1* ^*−/−*^ mice compared with WT mice at 2 dpi, whereas no significant differences were detected at the other time points (Figure S5). Similarly, neutrophil counts in BALF were significantly higher in *Pdcd1* ^*−/−*^ mice compared to WT controls at 2 dpi (*p* < 0.001; Figure 2A). Immunohistochemical staining for Ly6g revealed widespread positive signals in the airways, alveolar spaces, lung interstitium, and alveolar walls of *Pdcd1* ^*−/−*^ mice, with particularly prominent staining observed at 2 dpi (Figure 2B). Quantitative analysis confirmed that the proportion of Ly6g^+^ neutrophils was significantly greater in infected *Pdcd1* ^*−/−*^ mice than that in WT mice (Figure 2C). Consistent with this cellular infiltration, the pulmonary expression of the neutrophil chemoattractants *Cxcl1* and *Cxcl2* was markedly upregulated in *Pdcd1* ^*−/−*^ mice (*p* < 0.001, Figure 2D; *p* < 0.0001, Figure 2E). Furthermore, expression of myeloperoxidase (Mpo), a marker of neutrophil activation, was also significantly elevated in *Pdcd1* ^*−/−*^ mice (*p* < 0.0001; Figure 2F). Together, these data show that *Pdcd1* knockout enhances neutrophil recruitment, potentiates chemokine‐driven infiltration, and promotes neutrophil activation in the lung during acute RSV infection, which correlates with more severe disease manifestations.

**Figure 2:**
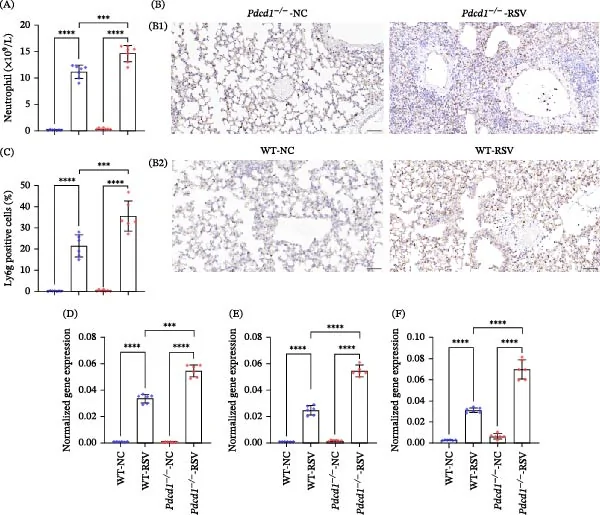
*Pdcd1* ^*−/−*^ mice exhibited increased neutrophil infiltration in the lungs during acute RSV infection. The lungs and bronchoalveolar lavage fluid (BALF) were collected at 2 dpi. (A) Neutrophil counts in the BALF (10^9^/L, *n* = 7 mice per group). (B) IHC staining for Ly6g^+^ neutrophils infiltration in the lungs of mice. The subfigure (B1) displays the lungs of *Pdcd1* ^*−/−*^ mice, while the subfigure (B2) shows the lungs of WT mice. Scale bars, 50 μm. (C) The proportion of Ly6g positive cells was quantified in six fields of view per slide, with a total of six slides analyzed. (D) The lungs of mice were homogenized to isolate total RNA. RT‐qPCR was performed for relative quantification of the mRNA expression of *Cxcl1*(D), *Cxcl2* (E), *Mpo* (F), *n* = 6 mice per group. Data represented means ± SD,  ^∗∗∗^ *p* < 0.001,  ^∗∗∗∗^ *p* < 0.0001 using a two‐tailed Student’s *t*‐test.

### 3.3. *Pdcd1* Knockout Drives Th2‐Biased CD4^+^ T Cell Polarization and Compromises CD8^+^ T Cell Cytotoxicity During Virus Infection

Given the more pronounced histopathological changes observed in *Pdcd1* ^*−/−*^ mice (Figure 1C,D), we investigated whether this effect was mediated by aberrant T‐cell responses due to *Pdcd1* knockout. At 4 dpi, flow cytometric analysis revealed that the frequency of pulmonary CD4^+^ T cells was significantly higher in *Pdcd1* ^*−/−*^ mice than in WT mice (1.31‐fold; *p* < 0.001; Figure 3A–C). Further subset analysis showed that *Pdcd1* ^*−/−*^ mice exhibited elevated IL‐4^+^ Th2 cell frequencies (1.40‐fold) and reduced IFN‐γ^+^ Th1 cell frequencies (1.59‐fold) compared with WT controls (Figure 3D–I), indicating a Th2‐biased polarization profile in the absence of *Pdcd1*.

Figure 3CD4^+^ T cell responses in the lungs of WT and *Pdcd1* ^*−/−*^ mice at 4 dpi. (A) Representative flow plots of CD4^+^ T cells (CD45^+^CD3^+^CD4^+^). (B, C) Quantification of the frequencies (B) and absolute numbers (C) of CD4^+^ T cells in the lungs. (D) Representative histograms showing IL‐4 expression in CD4^+^ T cells. (E, F) Frequencies and absolute numbers of Th2 (IL‐4^+^) cells among CD4^+^ T cells. (G) Representative histograms showing IFN‐γ expression in CD4^+^ T cells. (H, I) Frequencies and absolute numbers of Th1 (IFN‐γ^+^) cells among CD4^+^ T cells. Data are presented as mean ± SD (*n* = 5 mice per group). Statistical significance was determined by unpaired two‐tailed Student’s *t*‐test.  ^∗^ *p* < 0.05,  ^∗∗^ *p* < 0.01,  ^∗∗∗^ *p* < 0.001,  ^∗∗∗∗^ *p* < 0.0001; ns, not significant.

**Figure:**
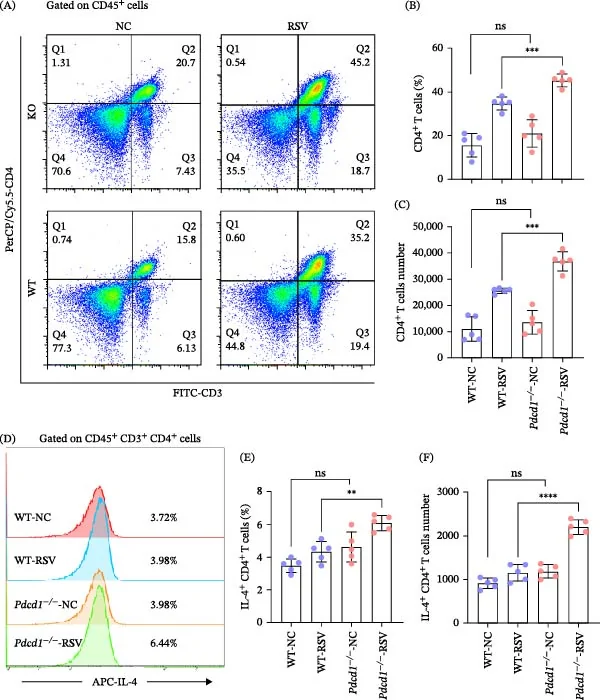

**Figure:**
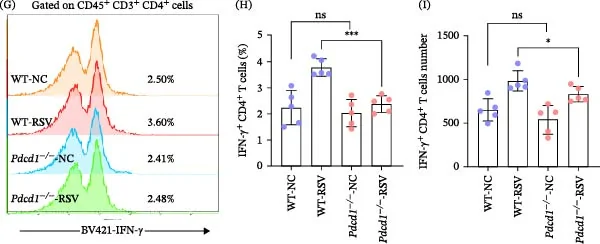

At 7 dpi, lymphocyte counts in BALF were significantly higher in *Pdcd1* ^*−/−*^ mice compared to WT controls (*p* < 0.001; Figure 4A). Immunohistochemical staining for CD3 revealed widespread positive signals in perivascular and peribronchiolar regions of the lungs (Figure 4B), and the proportion of infiltrating CD3^+^ T cells was significantly greater in *Pdcd1* ^*−/−*^ mice than that in WT mice (Figure 4C). Flow cytometry analysis of BALF and lung tissue suspensions at 7 dpi showed that RSV infection induced a higher frequency of CD4^+^ T cells in *Pdcd1* ^*−/−*^ mice compared to WT controls (Figure 4D). Quantitatively, CD4^+^ T cell frequency was 1.62‐fold higher in lung tissue and 1.85‐fold higher in BALF in *Pdcd1* ^*−/−*^ mice (*p* < 0.001, Figure 4E; *p* < 0.0001, Figure 4F). Further characterization of CD4^+^ T cell subsets revealed that the frequency of IL‐4^+^ (Th2) cells was elevated (1.63‐fold), whereas IFN‐γ^+^ (Th1) cells were reduced (1.92‐fold) in *Pdcd1* ^*−/−*^ mice compared to WT mice (*p* < 0.05, Figure 4G; *p* < 0.001, Figure 4H), further supporting a persistent Th2‐biased CD4^+^ T cell response in the absence of *Pdcd1*.

**Figure 4:**
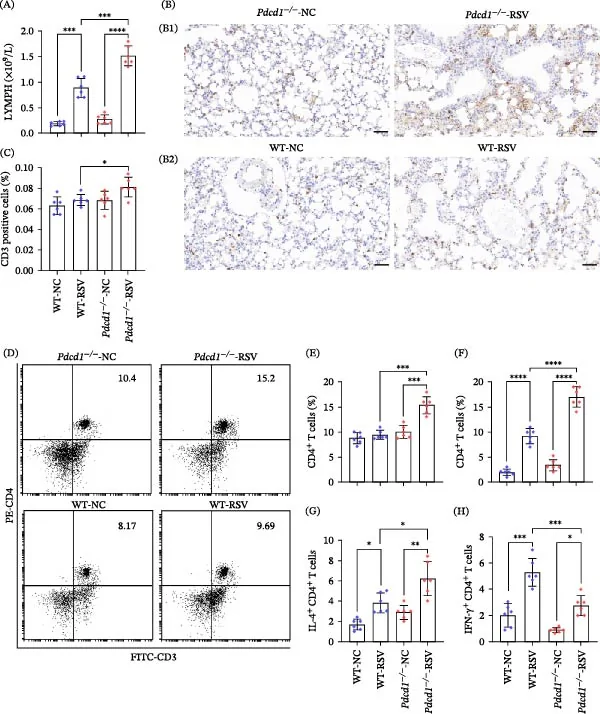
*Pdcd1* knockout promotes Th2‐biased CD4^+^ T‐cell polarization in the lung after RSV infection. Lungs and BALF were collected at 7 dpi. (A) Lymphocyte counts in the BALF (10^9^/L, *n* = 6 mice per group). (B) Representative images of CD3 immunohistochemical staining in lung sections from *Pdcd1* ^*−/−*^ (B1) and WT (B2) mice. Scale bars, 50 μm. (C) The proportion of CD3 positive cells was quantified in six fields of view per slide, with a total of six slides analyzed. Lung tissues from mice were processed into single‐cell suspensions, and the supernatant was analyzed by flow cytometry. (D) Representative flow plots of CD4^+^ T cells (CD45^+^CD3^+^CD4^+^). Frequencies of CD4^+^ T cells in lung (E) and BALF (F). Frequencies of IL‐4^+^ (Th2; G) and IFN‐γ^+^ (Th1; H) cells among CD4^+^ T cells. Data are mean ± SD; *n* = 6 mice per group.  ^∗^ *p* < 0.05,  ^∗∗^ *p* < 0.01,  ^∗∗∗^ *p* < 0.001,  ^∗∗∗∗^ *p* < 0.0001 using a two‐tailed Student’s *t*‐test.

In parallel, we characterized the CD8^+^ T‐cell response at both 4 and 7 dpi. The percentages and absolute numbers of CD8^+^ T cells were comparable between *Pdcd1* ^*−/−*^ and WT mice at both time points (Figure 5A–C and Figure 6A–C). However, functional assessment revealed that perforin and granzyme B secretion was markedly impaired in *Pdcd1* ^*−/−*^ mice, whereas IFN‐γ production remained comparable between the two genotypes (Figure 5D–L and Figure 6D–L), indicating an unresponsive CD8^+^ T cell effector phenotype. Collectively, these findings suggest that *Pdcd1* knockout promotes a Th2‐biased CD4^+^ T‐cell response while compromising CD8^+^ T‐cell cytotoxicity, which may contribute to impaired viral clearance and exacerbated immunopathology during RSV infection.

Figure 5CD8^+^ T cell responses in the lungs of WT and *Pdcd1* ^*−/−*^ mice at 4 dpi. (A) Representative flow plots of CD8^+^ T cells (CD45^+^CD3^+^CD8^+^). (B, C) Quantification of the frequencies (B) and absolute numbers (C) of CD8^+^ T cells in the lungs. (D) Representative histograms showing IFN‐γ expression in CD8^+^ T cells. (E, F) Frequencies and absolute numbers of IFN‐γ^+^ CD8^+^ T cells. (G) Representative histograms showing granzyme B expression in CD8^+^ T cells. (H, I) Frequencies and absolute numbers of granzyme B^+^ CD8^+^ T cells. (J) Representative histograms showing perforin expression in CD8^+^ T cells. (K, L) Frequencies and absolute numbers of perforin^+^ CD8^+^ T cells. Data are presented as mean ± SD (*n* = 5 mice per group). Statistical significance was determined by unpaired two‐tailed Student’s *t*‐test.  ^∗^ *p* < 0.05,  ^∗∗^ *p* < 0.01,  ^∗∗∗^ *p* < 0.001,  ^∗∗∗∗^ *p* < 0.0001; ns, not significant.

**Figure:**
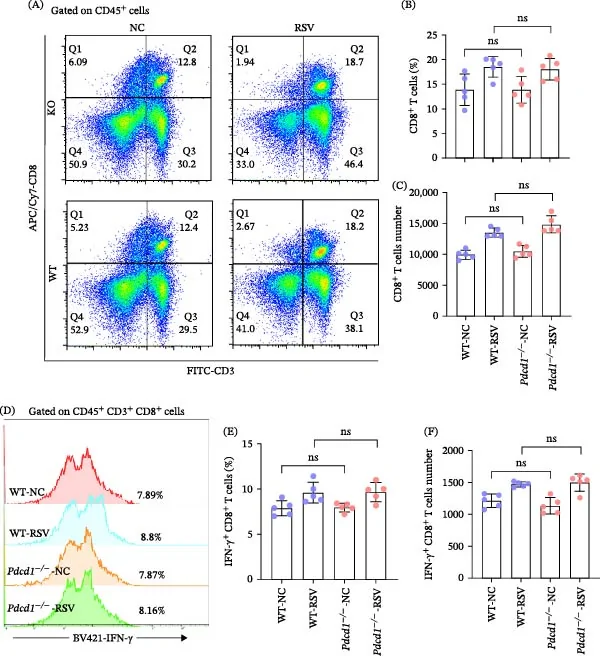

**Figure:**
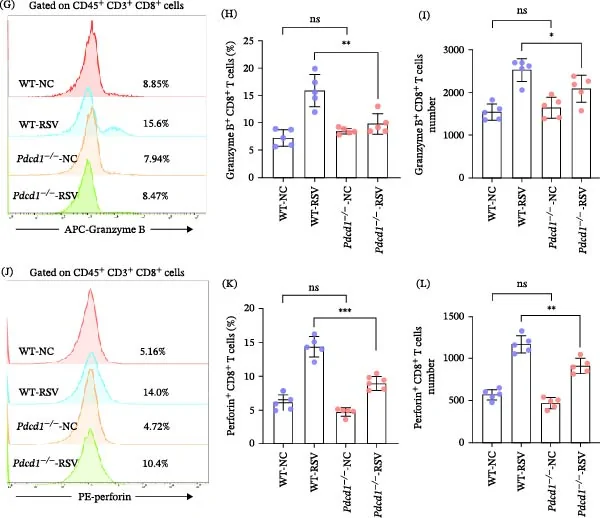

Figure 6CD8^+^ T cell responses in the lungs of WT and *Pdcd1* ^*−/−*^ mice at 7 dpi. (A) Representative flow plots of CD8^+^ T cells (CD45^+^CD3^+^CD8^+^). (B, C) Quantification of the frequencies (B) and absolute numbers (C) of CD8^+^ T cells in the lungs. (D) Representative histograms showing IFN‐γ expression in CD8^+^ T cells. (E, F) Frequencies and absolute numbers of IFN‐γ^+^ CD8^+^ T cells. (G) Representative histograms showing granzyme B expression in CD8^+^ T cells. (H, I) Frequencies and absolute numbers of granzyme B^+^ CD8^+^ T cells. (J) Representative histograms showing perforin expression in CD8^+^ T cells. (K, L) Frequencies and absolute numbers of perforin^+^ CD8^+^ T cells. Data are presented as mean ± SD (*n* = 5 mice per group). Statistical significance was determined by unpaired two‐tailed Student’s *t*‐test.  ^∗^ *p* < 0.05,  ^∗∗^ *p* < 0.01,  ^∗∗∗^ *p* < 0.001,  ^∗∗∗∗^ *p* < 0.0001; ns, not significant.

**Figure:**
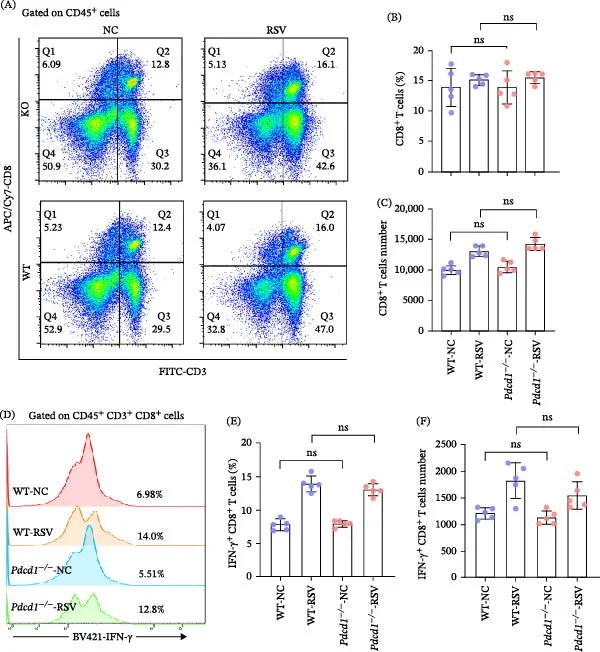

**Figure:**
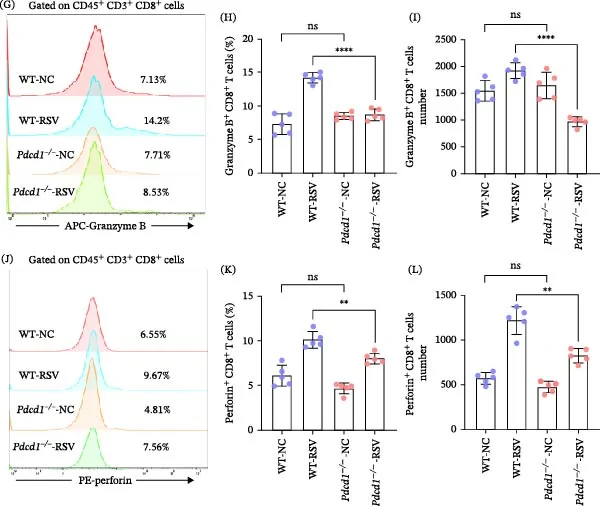

### 3.4. *Pdcd1* ^*−/−*^ Mice Showed a Significantly Reduced Antiviral Cytokine Secretion Following Virus Infection

Given the protective role of IFN‐γ and IFN‐α in limiting RSV severity [16, 17], we measured their expression in both lung tissue and the serum of infected mice. In the lungs, IFN‐γ expression was significantly lower in *Pdcd1* ^*−/−*^ mice than in WT controls at 2, 4, and 7 dpi (Figure 7A). Correspondingly, serum IFN‐γ levels were also markedly reduced in *Pdcd1* ^*−/−*^ mice at 4 dpi (Figure 7B). Similarly, pulmonary IFN‐α expression was significantly decreased in *Pdcd1* ^*−/−*^ mice at 7 dpi (Figure 7C), and serum IFN‐α levels were lower at 2 dpi compared to WT mice (Figure 7D).

**Figure 7:**
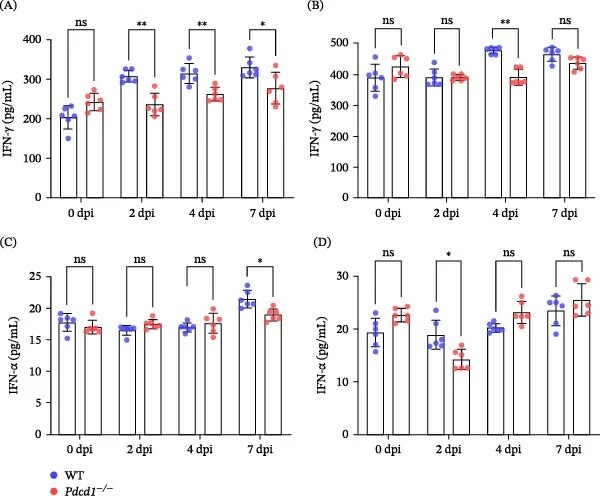
*Pdcd1* knockout reduces systemic and pulmonary interferon production after RSV infection. Lungs and serum were collected at indicated time points postinfection. IFN‐γ and IFN‐α levels were measured by ELISA. IFN‐γ concentrations in lung homogenates (A) and serum (B). IFN‐α concentrations in lung homogenates (C) and serum (D). Data are presented as mean ± SD (*n* = 6 mice per group). Statistical significance was determined by two‐way ANOVA followed by Tukey’s multiple comparisons test.  ^∗^ *p* < 0.05,  ^∗∗^ *p* < 0.01; ns, not significant.

Together, these data indicate that *Pdcd1* knockout leads to impaired production of key antiviral cytokines—both locally in the lung and systemically—following RSV infection, which may contribute to the enhanced susceptibility and exacerbated disease observed in *Pdcd1* ^*−/−*^ mice.

### 3.5. *Pdcd1* ^*−/−*^ Mice Also Exhibit Enhanced Susceptibility to the RSV A2 Strain

To determine whether the observed phenotype is strain‐specific or broadly applicable, we extended our analysis to an additional RSV strain, A2. Mice were inoculated i.n. with 10^6^ PFU of RSV A2, and disease progression was assessed by daily body weight monitoring, pulmonary viral load quantification (absolute copies of the RSV N gene), and histopathological evaluation via H&E staining. Consistent with the findings from the long strain experiments, *Pdcd1* ^*−/−*^ mice infected with RSV A2 exhibited significantly greater body weight loss (Figure 8A), higher pulmonary viral loads (Figure 8B), and more pronounced inflammatory cell infiltration compared with WT controls (Figure 8C,D). Collectively, these results demonstrate that *Pdcd1* ^*−/−*^ mice are highly susceptible to RSV infection, as observed with both the RSV Long and RSV A2 strains.

**Figure 8:**
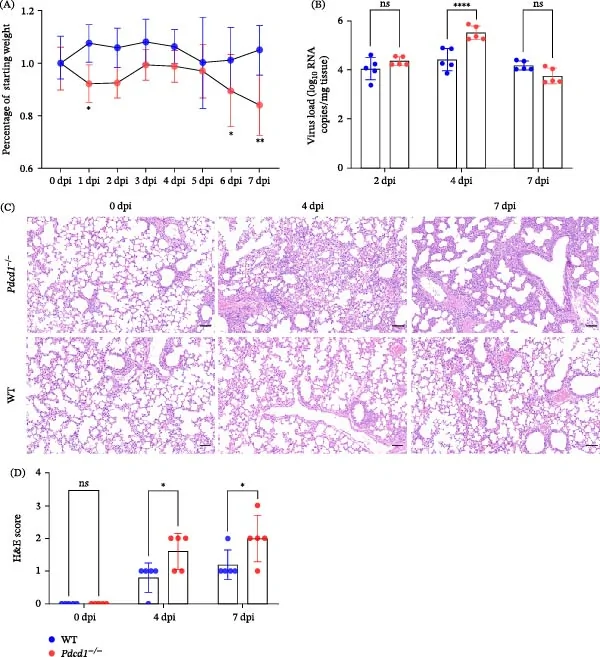
*Pdcd1* ^*−/−*^ mice are susceptible to intranasal RSV infection. Mice were inoculated intranasally with 10^6^ PFU of purified RSV A2 strain. (A) Body weight was monitored daily for 7 dpi. At the time of inoculation (0 dpi), the body weights of mice were set as the baseline, and subsequent weight changes relative to the baseline are shown (*n* = 3–5 mice per group). (B) Viral loads in the lungs of RSV‐infected mice were measured at the indicated time points. *n* = 5 mice per group. (C) Lungs were collected on 0, 4, and 7 dpi and subjected to H&E staining. Scale bars, 50 μm. (D) Statistical analysis based on histological scores for perivascular and alveolar wall inflammatory cell infiltration. *n* = 5 mice per group. All data are presented as mean ± SD. Statistical significance was determined by two‐way ANOVA with Tukey’s post hoc test.  ^∗^ *p* < 0.05,  ^∗∗^ *p* < 0.01,  ^∗∗∗∗^ *p* < 0.0001 versus WT mice; ns, not significant.

## 4. Discussion

This study provides the first systematic evidence that *Pdcd1* knockout disrupts the balance between innate and adaptive immunity during acute RSV infection, leading to severe viral pneumonia and establishing a novel, highly susceptible animal model. The *Pdcd1* ^*−/−*^ mouse phenotype—characterized by enhanced viral replication, significant pulmonary inflammation, an immunopathological signature of neutrophilic inflammation, Th2‐skewed CD4^+^ T‐cell responses, dysfunctional CD8^+^ T cells, and blunted interferon production—closely mirrors several critical features of severe RSV disease in human infants, particularly those in the youngest and most vulnerable populations [18].

The most salient finding of our work is the demonstration of immune dysregulation rather than mere immunodeficiency in *Pdcd1* knockout hosts. Unlike models of generalized immune compromise, *Pdcd1* ^*−/−*^ mice mount an exuberant but pathological response. The early, overwhelming neutrophil influx, supported by elevated CXCL1/CXCL2 and MPO, aligns with clinical observations linking neutrophilic inflammation to disease severity in infants with RSV bronchiolitis [19]. This suggests that PD‐1 plays a crucial early checkpoint role in restraining excessive innate inflammatory cell recruitment, possibly via modulating initial cytokine and chemokine circuits. Furthermore, the pronounced Th2 bias in the CD4^+^ T‐cell compartment provides a mechanistic link to the observed immunopathology. A Th2‐dominant environment is associated with mucus hypersecretion, eosinophil activation, and airway hyperresponsiveness in allergic asthma and viral exacerbations [20]. Our model thus recapitulates a key immune deviation observed in a subset of severe infant RSV cases, offering a platform to dissect how PD‐1 signaling normally constrains this pathogenic pathway.

Concurrently, we observed a state of CD8^+^ T‐cell dysfunction in *Pdcd1* ^*−/−*^ mice despite comparable cell numbers to WT controls. This “exhaustion‐like” impairment in an acute infection setting was evidenced by the significantly reduced expression of key cytolytic mediators—perforin and granzymes—which directly correlates with the higher pulmonary viral loads observed in *Pdcd1* ^*−/−*^ mice. Notably, however, viral loads were comparable between the two genotypes at 8 dpi, as reflected by similar RSV N gene copy numbers. This indicates that CD8^+^ T‐cell dysfunction primarily delayed, rather than completely abrogated, viral control. This finding is particularly intriguing given that PD‐1 is canonically understood to restrain T‐cell overactivation. Our data suggest that in its absence, CD8^+^ T cells may undergo aberrant activation or differentiation, leading to functional delay. This stands in contrast to chronic infection models, where PD‐1 upregulation is a hallmark of exhaustion, highlighting a context‐dependent role for PD‐1 in shaping CD8^+^ T‐cell functional quality during acute respiratory viral infection.

The significant impairment in IFN‐γ and IFN‐α production consolidates the model’s susceptibility. IFN‐γ is vital for antiviral defense and macrophage activation [21], while type‐I interferons are central to establishing the initial antiviral state [22]. Their combined deficiency in *Pdcd1* ^*−/−*^ mice creates a permissive environment for RSV replication and likely contributes to the skewed adaptive response. This blunted interferon axis mirrors the poorly developed interferon response observed in young infants [23], further strengthening the translational relevance of our model.

While our study strongly positions the *Pdcd1* ^*−/−*^ mouse as a superior model for severe RSV, certain limitations should be noted. The model does not fully replicate all aspects of human infant physiology, such as lung development and the naive state of the infant adaptive immune system. Furthermore, the precise cellular targets and downstream signaling pathways through which PD‐1 exerts its regulatory effects during the RSV infection warrant further investigation. Future studies employing cell‐specific *Pdcd1* knockouts or in vivo blockade experiments will be crucial to dissect whether the phenotype is driven primarily by T‐cell‐intrinsic mechanisms or by dysregulation in antigen‐presenting cells.

## 5. Conclusions

We have demonstrated that PD‐1 is a critical regulator of immunopathology during RSV infection. Its absence leads to a coherent and severe disease phenotype driven by dysregulated innate inflammation, biased adaptive immunity, and deficient antiviral cytokine responses. The *Pdcd1* ^*−/−*^ mouse model, by more accurately reflecting the immune determinants of severe human RSV disease, provides a valuable and sensitive preclinical tool. It will be particularly useful for evaluating the efficacy of novel immunomodulatory therapies, next‐generation vaccines, and antiviral agents aimed at preventing or treating severe RSV in high‐risk populations.

## Funding

This work was supported by the Natural Science Basic Research Program of Shaanxi Province (Grant 2025JC‐YBQN‐1178) and the Special Project for the Cultivation and Promotion of Young Physicians in Xijing Hospital (Grant XJZT25QN02). Ruixue Ma was in receipt of these grants.

## Ethics Statement

All animal experiments were approved by the Medical Ethics Committee of the First Affiliated Hospital of the Air Force Medical University (Number 20250076).

## Conflicts of Interest

The authors declare no conflicts of interest.

## Supporting Information

Additional supporting information can be found online in the Supporting Information section.

## Supporting information


**Supporting Information 1** Table S1: Primer sequences and PCR conditions for *Pdcd1* genotyping in mice. The table lists the forward and reverse primer sequences, expected product sizes, and annealing temperatures used for the two primer sets (primers 1 and 2) to identify the *Pdcd1* genotype.

**Supporting Information 2** Table S2: Primer sequences for target genes used in RT‐qPCR. The table lists the forward and reverse primer sequences for the target genes (*Cxcl1*, *Cxcl2*, *Mpo*, *Prf1*, *Gzma*, *Gzmb*, *Lta*, and *RSV N*) and the reference gene (*Actb*) used for RT‐qPCR analysis.

**Supporting Information 3** Figure S1: Generation and genotypic verification of *Pdcd1^−/−^* mice. (A) Schematic diagram of the targeting strategy for the *Pdcd1* gene. (B) Representative agarose gel electrophoresis of PCR genotyping products. Expected band sizes: homozygous knockout, 416 bp; heterozygous, 416 , 766 , and 2931 bp; wild‐type (WT), 766 and 2931 bp. The presence of the expected bands confirmed the correct genotypes.

**Supporting Information 4** Figure S2: Gating strategy for neutrophils. The schematic outlines the identification of neutrophils from lung samples and the subsequent analysis of their surface markers. Lymphocytes were initially gated based on FSC‐A and SSC‐A, followed by singlet gating using FSC‐A and FSC‐H. The CD45^+^ subset was then selected, and neutrophils were identified as CD11b^+^ Ly6g^+^ cells.

**Supporting Information 5** Figure S3: Gating strategy for CD4^+^ T cells and their intracellular cytokines. The schematic outlines the identification of CD4^+^ T‐cell subsets and the subsequent analysis of their respective intracellular cytokines from lung samples. After gating on the CD45^+^ population, CD4^+^ T cells were identified as CD3^+^ CD4^+^ cells, and the expression of IL‐4 and IFN‐γ was subsequently analyzed within this gated population.

**Supporting Information 6** Figure S4: Gating strategy for CD8^+^ T cells and their intracellular cytokines. The schematic outlines the identification of CD8^+^ T‐cell subsets and the subsequent analysis of their respective intracellular cytokines from lung samples. After gating on the CD45^+^ population, CD8^+^ T cells were identified as CD3^+^ CD8^+^ cells, and the expression of IFN‐γ, granzyme B, and perforin was subsequently analyzed within this gated population.

**Supporting Information 7** Figure S5: Flow cytometric analysis of pulmonary neutrophil infiltration in WT and *Pdcd1^−/−^* mice after RSV infection. (A) Representative flow cytometry plots of neutrophils (CD45^+^CD11bb^+^Ly6g^hi^) in mouse lungs cells. Quantification of the frequencies (B) and absolute numbers (C) of the neutrophils. All data are presented as mean ± SD. Statistical significance was determined by two‐way ANOVA with Tukey’s post hoc test.  ^∗^ *p* < 0.05,  ^∗∗∗∗^ *p* < 0.0001 versus WT mice. *n* = 5 mice per group.

## Data Availability

Data will be made available upon request.
