# Supplementary material for: Pdcd1−/− Mice Exhibit High Susceptibility to Respiratory Syncytial Virus Infection
Source: J Immunol Res. 2026 Oct 22;2026:1223280. doi: 10.1155/jimr/1223280 (PMC13691424; doi:10.1155/jimr/1223280)
Supplement: Supplementary file 1 [file JIMR-2026-1223280-s001.docx]

**Table S1. Primer sequences and PCR conditions for *Pdcd1* genotyping in mice.**

|  | **Forward primer sequences (5’→3’)** | **Reverse primer sequences (5’→3’)** | **Product size**  **(bp)** | **Annealing Temperature (℃)** |
| --- | --- | --- | --- | --- |
| Primers 1 | GCCAAATGACTACATCGGCTAAA | CATAATCTCAACCCTTCCCTATGC | 416 | 60 |
| Primers 2 | CCAGGAACTCCCCGTTAGTAAATG | CATAATCTCAACCCTTCCCTATGC | 766 | 60 |
