# Supplementary material for: Pdcd1−/− Mice Exhibit High Susceptibility to Respiratory Syncytial Virus Infection
Source: J Immunol Res. 2026 Oct 22;2026:1223280. doi: 10.1155/jimr/1223280 (PMC13691424; doi:10.1155/jimr/1223280)
Supplement: Supplementary file 2 [file JIMR-2026-1223280-s002.docx]

**Table S2. Primer sequences for target genes used in RT-qPCR.**

| **Target genes** | **Forward primer sequences (5’→3’)** | **Reverse primer sequences (5’→3’)** |
| --- | --- | --- |
| *Cxcl1* | CTGGGATTCACCTCAAGAACATC | CAGGGTCAAGGCAAGCCTC |
| *Cxcl2* | CCAACCACCAGGCTACAGG | GCGTCACACTCAAGCTCTG |
| *Mpo* | AGTTGTGCTGAGCTGTATGGA | CGGCTGCTTGAAGTAAAACAGG |
| *Prf1* | AGCACAAGTTCGTGCCAGG | GCGTCTCTCATTAGGGAGTTTTT |
| *Gzma* | TGCTGCCCACTGTAACGTG | GGTAGGTGAAGGATAGCCACAT |
| *Gzmb* | CCACTCTCGACCCTACATGG | GGCCCCCAAAGTGACATTTATT |
| *Lta* | CCACCTCTTGAGGGTGCTTG | CATGTCGGAGAAAGGCACGAT |
| *RSV N* | CATCCAGCAAATACACCATCCA | TTCTGCACATCATAATTAGGAGTATCAA |
| *Actb* | GGCTGTATTCCCCTCCATCG | CCAGTTGGTAACAATGCCATGT |
