# Supplementary material for: Pdcd1−/− Mice Exhibit High Susceptibility to Respiratory Syncytial Virus Infection
Source: J Immunol Res. 2026 Oct 22;2026:1223280. doi: 10.1155/jimr/1223280 (PMC13691424; doi:10.1155/jimr/1223280)
Supplement: Supplementary file 3 [file JIMR-2026-1223280-s003.docx]

**Figure S1. Generation and genotypic verification of *Pdcd1^-/-^* mice.** (A) Schematic diagram of the targeting strategy for the *Pdcd1* gene; (B) Representative agarose gel electrophoresis of PCR genotyping products. Expected band sizes: homozygous knockout, 416 bp; heterozygous, 416 bp, 766 bp, and 2,931 bp; wild-type (WT), 766 bp and 2,931 bp. The presence of the expected bands confirmed the correct genotypes.
