# Supplementary material for: Pdcd1−/− Mice Exhibit High Susceptibility to Respiratory Syncytial Virus Infection
Source: J Immunol Res. 2026 Oct 22;2026:1223280. doi: 10.1155/jimr/1223280 (PMC13691424; doi:10.1155/jimr/1223280)
Supplement: Supplementary file 4 [file JIMR-2026-1223280-s004.docx]

**Figure S2. Gating strategy for neutrophils.** The schematic outlines the identification of neutrophils from lung samples and the subsequent analysis of their surface markers. Lymphocytes were initially gated based on FSC-A and SSC-A, followed by singlet gating using FSC-A and FSC-H. The CD45⁺ subset was then selected, and neutrophils were identified as CD11b⁺ Ly6G⁺ cells.
