# Supplementary material for: Pdcd1−/− Mice Exhibit High Susceptibility to Respiratory Syncytial Virus Infection
Source: J Immunol Res. 2026 Oct 22;2026:1223280. doi: 10.1155/jimr/1223280 (PMC13691424; doi:10.1155/jimr/1223280)
Supplement: Supplementary file 5 [file JIMR-2026-1223280-s005.docx]

**Figure S3. Gating strategy for CD4^+^ T cells and their intracellular cytokines.** The schematic outlines the identification of CD4⁺ T-cell subsets and the subsequent analysis of their respective intracellular cytokines from lung samples. After gating on the CD45⁺ population, CD4⁺ T cells were identified as CD3⁺ CD4⁺ cells, and the expression of IL-4 and IFN-γ was subsequently analyzed within this gated population.
