# Supplementary material for: Pdcd1−/− Mice Exhibit High Susceptibility to Respiratory Syncytial Virus Infection
Source: J Immunol Res. 2026 Oct 22;2026:1223280. doi: 10.1155/jimr/1223280 (PMC13691424; doi:10.1155/jimr/1223280)
Supplement: Supplementary file 6 [file JIMR-2026-1223280-s006.docx]

**Figure S4. Gating strategy for CD8^+^ T cells and their intracellular cytokines.** The schematic outlines the identification of CD8⁺ T-cell subsets and the subsequent analysis of their respective intracellular cytokines from lung samples. After gating on the CD45⁺ population, CD8⁺ T cells were identified as CD3⁺ CD8⁺ cells, and the expression of IFN-γ, Granzyme B and Perforin was subsequently analyzed within this gated population.
