# Supplementary material for: Pdcd1−/− Mice Exhibit High Susceptibility to Respiratory Syncytial Virus Infection
Source: J Immunol Res. 2026 Oct 22;2026:1223280. doi: 10.1155/jimr/1223280 (PMC13691424; doi:10.1155/jimr/1223280)
Supplement: Supplementary file 7 [file JIMR-2026-1223280-s007.docx]

**Figure S5. Flow cytometric analysis of pulmonary neutrophil infiltration in WT and *Pdcd1^-/-^* mice after RSV infection.** (A) Representative flow cytometry plots of neutrophils (CD45^+^CD11b^+^Ly6g^hi^) in mouse lungs cells. Quantification of the frequencies (B) and absolute numbers (C) of the neutrophils. All data are presented as mean±SD. Statistical significance was determined by two-way ANOVA with Tukey's post-hoc test. **P* < 0.05, *****P* < 0.0001 versus WT mice. n = 5 mice per group.
